# Experiencing and navigating occupational stigma as a peer support worker in mental health services: a qualitative exploration

**DOI:** 10.1186/s13033-026-00712-1

**Published:** 2026-05-08

**Authors:** Trishna Chauhan

**Affiliations:** School of Medicine and Dentistry, The University of Lancashire, Preston, Lancashire UK

**Keywords:** Peer support workers, Mental health, Stigma

## Abstract

**Background:**

Peer support workers (PSWs) provide support to others through their personal lived experiences of mental health. However, their work is often undervalued by their colleagues, and they frequently face challenges in the workplace, resulting in occupational stigma. Currently, there are limited insights into how PSWs experience and manage the stigma they face. Therefore, this study examines how PSWs in the UK National Health Service experience and navigate occupational stigma in their roles.

**Methods:**

Seventy semi-structured interviews were conducted with PSWs and their colleagues. Interviews explored their experiences in the role, workplace interactions, and subsequently perceptions and experiences of stigma, and how they dealt with stigmatising experiences. The data were analysed using thematic analysis to identify how stigma manifested and how they navigated it.

**Results:**

PSWs reported experiencing stigma both covertly and explicitly. Covert stigma included subtle devaluation of their knowledge and exclusion from decision-making, while explicit stigma involved direct questioning of competence and disrespectful behaviour from colleagues. In response, PSWs navigated stigma through three main strategies. First, they demonstrated commitment to their role via reliability, dedication, and consistent performance, reinforcing the value of their work. Second, PSWs leveraged experiential knowledge as expertise, emphasising practical skills and lived experience in patient care. Third, they used their roles to create reciprocal benefits, where they supported service-users, which in turn helped their own mental health and recovery.

**Conclusion:**

Occupational stigma towards PSWs is pervasive, manifesting in both subtle and overt ways that can undermine their role. PSWs actively counter stigma through commitment, expertise, and reciprocal relationships, highlighting their resilience and adaptability. Addressing stigma in healthcare settings is critical for improving team dynamics and ensuring high-quality care. Going forward to support the role, policymakers and organisations that employ PSWs should focus on improving organisational culture, recognition of the role, and collaborative practices to reduce stigma, strengthen workforce sustainability and recognise the value of lived experience in the workforce.

## Introduction

Stigma has been consistently pervasive in mental health [1, 2]. Despite growing public awareness and anti-stigma campaigns [3], individuals with mental health diagnoses continue to experience negative stereotyping, discrimination, and social exclusion [4, 5, 6]. The recovery movement in mental health emerged in response to the limitations of the traditional medical model, which often framed mental illness in terms of chronicity, incapacity, and dependence [7]. In turn, it directly challenges stigma by promoting person-centred narratives [8], based on service-user activism which advocates that individuals living with mental health challenges can lead meaningful, autonomous lives even in the presence of ongoing symptoms [9, 10]. Central to the movement is a shift away from viewing individuals as passive recipients of care, toward seeing them as active agents in their own recovery [9]. This reconceptualisation has also played a role in challenging stigma by positioning people with lived experience as credible, resilient, and valuable contributors to both mental health services and broader society [11].

A key innovation arising from the recovery-based approach is the formal integration of Peer Support Workers (PSWs) into mental health services (e.g. [12–16]). PSWs are employed specifically because of their lived experience of mental health and, often, mental health service use as an inpatient and/or service-user. Their occupational role is grounded in the recovery ethos, offering relational, experiential support to current service-users and modelling hope and possibility. In doing so, PSWs seek to actively contest the stigma traditionally associated with mental illness by reframing lived experience as a source of knowledge and expertise [17].

The presence of PSWs thus represents a strategic unmasking of a highly stigmatised identity within organisational settings. Rather than concealing their mental health histories, PSWs leverage these experiences to challenge stereotypes, support service-users, work complementarily to clinical professionals, and promote recovery [12, 16]. Extant research highlights the multiple benefits of PSWs [18, 19, 20]. For example, Tse et al. [20] found service-users’ somatic symptoms improved and were able to connect better with others, following time spent with PSWs. In their review, Cooper et al. [18] reported that although the PSW role is often ill-defined, it can still be effective at improving some clinical outcomes, self-efficacy and promoting recovery. In addition, by employing PSWs, healthcare organisations are able to demonstrate inclusivity for recovery-oriented approaches [21].

However, the role is not without its challenges and tensions. Some scholars have noted the risk of professionalisation, whereby the original grassroots ethos of peer support is diluted by formalised training, standardisation, and alignment with clinical norms [22, 23]. Additionally, PSWs may face power imbalances within mental health teams, where their contributions are devalued [24]. Often, these dynamics place PSWs in liminal positions: neither fully clinicians nor entirely service-users, causing tensions between their occupational role and their personal sense of self [25]. These negative experiences are often turbulent for PSWs, where they feel undermined in their role [26].

While stigma has been widely examined in relation to mental illness and marginalised populations [27, 28, 29], there is comparatively limited scholarly attention on the occupational stigma experienced by PSWs. Occupational stigma refers to the negative social judgements attached to a particular occupational role or its incumbents, whereby the work itself, or the identity it signals, is perceived as discrediting [30]. Unlike stigma directed at an individual’s personal characteristics, occupational stigma is structurally embedded, meaning that merely *occupying* a role can render a worker vulnerable to devaluation, regardless of their conduct [31, 32]. For PSWs, this dynamic is compounded such that their role is explicitly premised on a stigmatised identity (lived experience of mental illness), meaning they are uniquely exposed to stigma at both the individual and occupational level.

This paper addresses the need to better understand PSWs experience of occupational stigma and how they navigate it. The occupational stigma faced by PSWs can undermine their legitimacy, compromise their wellbeing, and limit their effectiveness within their role [33]. Understanding their experiences of stigma and how they navigate it is essential to inform policy, improve workforce integration, and promote inclusive workplace cultures. By exploring how PSWs experience occupational stigma and subsequently respond to it, this research contributes towards understanding how stigma impacts the PSW role, and its impact on the advancement of recovery-oriented systems that value lived experience as a form of expertise.

## Methodology

This research is situated in the National Health Service (NHS), involving 70 interviews with PSWs and their colleagues. A qualitative approach was used to interpret phenomena in terms of the meanings people bring to them [34]. As scholars have argued that stigmatising processes involve the behaviours, practices and discourse of others beyond the stigmatised group [29], interviews therefore included both PSWs and their colleagues who worked alongside them. The final sample consisted of 36 PSWs and 34 other employees (senior managers, organisational managers and clinical professionals) who worked alongside PSWs (see Table 1). The PSWs recruited had been diagnosed with a range of mental health conditions, including psychosis, schizoaffective disorder, personality disorder, depression, anxiety, post-natal depression, previous suicide attempts, obsessive-compulsive disorder and addiction. They were also recruited from a range of inpatient and ward-based settings.

**Table 1:** Participant sample via job roles

Job role	Number of respondents	Male / Female
Peer support worker (PSW)	36	M: 11 F: 25
Clinical professional (CP)	19	M: 6 F:13
Organisational manager (OM)	7	M: 2 F: 5
Senior manager (SM)	8	M: 2 F:6

A semi-structured interview guide was devised and utilised, enabling participants to respond freely in their own words and allowing follow-up questions to emerge. All interviews were audio-recorded and lasted between 20 min and two hours (average: 50 min). While most interviews were conducted over the phone, some were conducted face-to-face.

The original focus of the study was the experiences of PSWs in their role more broadly, including their workplace relationships, integration into teams, and day-to-day challenges. In the interviews with PSWs, the focus was on understanding how they experienced their role, their interactions and relationships with their colleagues and the extent to which they felt part of the organisation. They were specifically asked about the challenges that they encountered in their role and how they navigated these challenges. This is where individuals disclosed their experiences of stigma, which became the phenomenon of interest for this study. Stigma was not an anticipated focus; rather, it emerged inductively and unprompted across interviews as a consistent and prominent theme in participants’ accounts. This inductive emergence of stigma as the central phenomenon reflects the qualitative, exploratory nature of the study, and its dominance across the data warranted the focused analysis presented here.

Other employees were asked about their perceptions of the PSW role and role occupants, the challenges encountered by PSWs and how these are negotiated. It also allowed further exploration of how other employees witnessed and discussed occupational stigma experienced by PSWs. Interviewing other employees became a useful mechanism for triangulating the voices of multiple actors [35]. For example, some PSWs described incidents of not being treated like a member of staff and line managers of these PSWs corroborated such accounts by commenting on how PSWs were excluded from meetings because of having previously been a patient or an ongoing service-user.

### Data analysis

Data was analysed using principles of thematic analysis [36]. First, how stigma manifested for PSWs was identified, including covert and overt forms through patronisation, exclusion and direct derogation. Second, how PSWs dealt with this stigma was identified through three main strategies: (i) demonstrating commitment (ii) leveraging experiential expertise and (iii) using the role for reciprocal benefits. Alongside this, how PSWs’ colleagues described their accounts were also included, in which similarities and divergences emerged in the stigmatising experiences.

## Reflexivity

The author of this manuscript deliberately positioned themselves as a novice to the subject matter, without personal lived experience of mental health or direct professional experience of peer support working. This was a conscious methodological choice to minimise the imposition of prior interpretations onto participants’ accounts, and to allow the meanings and experiences described by participants to guide the analysis. While this positionality reduced the risk of over-identification with participants, the researcher remained attentive to the assumptions that any researcher brings to qualitative inquiry. To manage this, reflective journaling was undertaken throughout data collection and analysis to document interpretive decisions and emerging preconceptions. Supervision meetings with doctoral supervisors provided an additional critical check on the analytical process.

In addition, to enhance credibility, the findings were shared with participants via member checking, and presented at a learning exchange attended by academics, service-users, and practitioners, whose responses were used to interrogate and refine the emerging analysis. Participants were also able to read their own transcript once transcribed to ensure that they were happy with that they said in the interview. A summary report was also returned to participating sites for review. Together, these strategies supported a transparent and critically reflexive approach to the research.

## Results

### Stigmatising experiences

PSWs experienced a range of stigmatising behaviours towards them, some of which manifested covertly, whereas others were more overt. Some PSWs reported that their experiences of stigma were directed towards their mental health condition: *“‘That’s just your personality disorder.’ It’s just that you’re not taken seriously and that’s rubbish.”* (PSW, #11). Other PSWs complained that their ability to do the job was explicitly questioned when they attempted to modify some of the practices on the ward: “*Every time I did try and say I’m not happy with this*,* or can you give me some advice*,* it was ‘well maybe you’re not well enough to this job’”* (PSW, #29). Several PSWs were also excluded from staff meetings, despite being in a formally paid role: “*Staff weren’t even sure they could let me in the office even though I am an employed member of staff*” (PSW, #29). Clinical professionals acknowledged these exclusionary practices: “*PSWs seem to be excluded from some of the stuff on the wards that the rest of the staff are doing*,* including being excluded from some of the meetings that they have on the wards*” (CP, #18), however, they failed to do anything to support the PSWs, reinforcing the exclusionary practices.

Covert forms of stigma were experienced through being patronised by staff in the workplace: *“People try and look after you and say: ‘Do you want this and that?’ And I’m like: ‘I’m not a child*,* you know I’m a grown adult’. It’s like they have to look after you*,* and it’s like: ‘No! I’m at work. I’m working*,* like you*.’” (PSW, #26). Rather than perceiving it as helpful, several PSWs found it overbearing, feeling like they were “*wrapped in cotton wool*” (PSW, #31). However, staff did not perceive this as a form of negative stigmatising behaviour, but rather being protective of PSWs: *“I’m probably being protective as well*,* – what happens if the person becomes unwell or if the job’s too much for them?”* (SM, #6). Yet most PSWs explicitly found it patronising: *“There have been a few times where my supervisor has tried to protect me from things and not told me about patients struggling and stuff like that… which I felt a little bit patronised”* (PSW, #20).

Moreover, comments were made by staff to other members of staff about PSWs that were negative: “*I heard some staff say stuff around like ‘I’m going to have to start locking up my purse’. ‘I’m going to have to get a locked drawer and start locking my things away’.”* (OM, #6). Indeed some senior managers, particularly those who were advocates of the role found other staff members to be resistant of the role: *“some people who just cannot accept the idea of having those users working alongside you*,* as part of a team member and will create difficult dynamics in the team to make the PSW feel out of place or not to make them feel included or kind of create friction”* (SM, #7).

Taken together, these experiences for PSWs were stigmatising for them and their role. In response to these experiences, PSWs took it upon themselves to navigate the stigma they experienced. As noted above, three strategies were identified in which PSWs utilised to navigate these stigmatising experiences to be able to perform their organisational role.

## Strategies for navigating stigma

### Demonstrating commitment

One strategy PSWs employed to navigate the stigma was by demonstrating commitment to their role. PSWs explicitly demonstrated commitment through a strong sense of timeliness and strict adherence to organisational rules and expectations. PSWs explained how they went out of their way to exceed expectations in the workplace as a way of challenging perceptions that people with mental health struggles at work: “*I present myself as an employee and as a member of staff. I am punctual and professional. I do my work in a timely fashion. I exceed myself in my daily duties. And so*,* I’m thinking*,* so what is it that makes me less than? It’s not how I conduct myself at work. It’s not how I go about doing my work. It’s purely the fact that I have got a mental health problem*,* or I have had mental health and used mental health services in the past.”* (PSW, #15) Moreover, PSWs found they constantly need to go above and beyond to reassure their colleagues about their ability to adhere to basic organisational rules: “*I do worry a little bit that people see me as a service-user - that needs reassuring a lot.*” (PSW, #2).

Indeed, this sense of proving themselves led to several PSWs overworking:*“Other people had said to me ‘She’ll not do it’ because I’ve been quite ill in the past. ‘She’ll never do it. She’ll not manage*,* shouldn’t have these peer workers…’ I proved them all wrong*,* but that’s part of me*,* cos if I hear something like that*,* I just go ‘Right. Sod you*,* I’m going to do it.’ And you know*,* for five years*,* I haven’t had one day off for five years*.*”* (PSW, #26). For some PSWs, this involved working harder: “*I felt that I had to work even harder than if I’d just been a normal support worker because I felt I had something to prove.”* (PSW, #30) which also created a detriment to their own health: “*I felt like I had to from the minute I stepped onto the ward to the minute I went home*,* that I had to prove I was worthy of being there. I just went above and beyond with the staff*,* and I did a lot of their work for them because I wasn’t treated very nicely. I really wanted to be liked. And so*,* I’d be like ‘oh I’ll do this for you*,* I’ll do that for you’ and ended up burning myself out*.” (PSW, #29). This was reiterated in the managerial interviews where they acknowledged that PSWs are: “*wanting to prove they are now well… Even if they have a cold or flu or something*,* they will still come to work*,* try just try and prove themselves because if they do take time off sick for any kind of*,* you know*,* your regular flu*,* stomach upset and stuff*,* people assume that they are sick because they’ve got mental health conditions*” (SM, #7).

Together, PSWs proving themselves in this way were aiming to challenge the misconceptions about their capabilities in order to build their credibility and respect in the organisation. However, the constant need to prove themselves often led to emotional exhaustion, overwork, and burnout, as PSWs felt pressured to exceed expectations to gain acceptance within traditional service structures.

## Leveraging experiential knowledge as expertise

In contrast to proving themselves, PSWs leveraged their experiential knowledge of mental health as a form of invaluable expertise that no other staff member could use. They often explicated their mental health as ‘expertise’: “*I have got lived experience; I have got expertise.*” (PSW, #35). As one PSW explains: “*We have lived so much of our life with mental health that we are*,* you know*,* in some ways very qualified for it.”* (PSW, #31), because “*You don’t go to university*,* and get your degree in being a peer worker*,* or in having the lived experience.*” (PSW, #33). Positioning their mental health as a form of expertise arguably contributes towards dispelling the stigma of mental health and helps PSWs with the purpose of their role.

Some PSWs highlighted their invaluable experiential expertise from lived experience that was an asset to them. One PSW explained that he was often complimented in his role because of his lived experience, he said people told him: *“‘Oh It’s really great what you’re doing because you’ve got lived experience’. And I know*,* it’s good. And now it’s like an asset to me now.”* (PSW #8). For others, they felt they could compare their expertise to clinical professionals to demonstrate how it is different yet complementary to professionals: “*You’ve got your unique skills and experience. But you’ve also got some understanding which perhaps other people don’t have…When I do groups with the psychologist*,* I can talk about examples from my own experience*,* whereas she can’t*.” (PSW, #11). For some, this led to positioning themselves as a professional: “*We’re professionals as Peer Support Workers”* (PSW, #47), because of how they used their lived experience in the organisation: “*So*,* it’s just tapping into it correctly. So*,* yeah*,* I feel quite strongly that it’s a professional role in that sense really*” (PSW #31).

Together, their positioning of their experiential expertise can be seen as a way of challenging the occupational stigma that undermines their role. By drawing on their lived experiences, they not only support other service-users with their expertise but in turn can educate colleagues about recovery-oriented perspectives and demonstrate the value of experiential knowledge within professional settings.

## Using the role for reciprocal benefits

Finally, PSWs took reciprocal benefits from the role, despite being stigmatised, because they used the role to help their own ongoing mental health recovery journey: “*Using your experience definitely helps you in your own recovery. So*,* I think that might be from a slightly selfish angle*,* it helps your own recovery as well as helping somebody else’s. But that’s the role*,* isn’t it?*” (PSW, #10). PSWs stated that this enabled them to realise the potential of how their illness could actually be considered a form of wellness: *“I can use my experiences to help other people. And it makes me feel a little bit less shit frankly”* (PSW, #27). The reciprocity in the role was described by other PSWs as relating to service-users through empowerment and empathy to help one’s own recovery: “*I’ve realised when I look back at my recovery path*,* what has helped me is getting involved with service-users and running groups. And empowering each other and having empathy for each other*.” (PSW, #12).

At the same time, PSWs also inspired current service-users and inpatients. PSWs highlighted how they were able to act as a role model to others, in supporting them through their lived experience of mental health: *“Some guy [patient]*,* grabbed hold of one of the psychologists and said*,* ‘until I heard Jerry [PSW*,* talking about himself] speak*,* I’ve never believed I could recover.’ And he said*,* hearing PSWs speak has made me think*,* well*,* if he can do it*,* I can do it*.” (PSW, #5). Other PSWs similarly emphasised how they can leverage their lived experience of mental health to serve as a form of hope for current service-users: “*Knowing that you’re inspiring them and offering them some hope that things will get better one day*.” (PSW, #23). Managers described PSWs as “*role models of possibility*” (SM, #5) who promoted: “*some kind of hope through peer support – ‘your life could be like this*.’” (SM, #6).

Taken together, the reciprocity of the role reinforced their sense of purpose within the organisation and helped to counteract feelings of marginalisation and affirming the value of lived experience as well as supporting their own ongoing recovery.

## Discussion

This study contributes to the growing body of literature exploring the role of PSWs, with a specific focus on their experiences of occupational stigma and how they navigate this in their workplace. The findings illustrate that PSWs occupy a complex position within organisational hierarchies; they are simultaneously celebrated as symbols of recovery yet constrained by enduring scepticism toward lived experience as a legitimate form of knowledge [37, 38]. As such, PSWs seek to carve out their role by overcoming the occupational stigma they face, by employing their own strategies to tackle this. This research identifies ways in which stigma is navigated in order for PSWs to perform their role, and in doing so, engage in an epistemic resistance that challenges dominant biomedical and professional paradigms [39, 40], prioritising their lived experience.

### Experiences of occupational stigma

PSWs described being patronised by colleagues in ways that were often subtle and couched in a language of concern or protectiveness. This covert form of stigma, where PSWs were treated as emotionally fragile or incapable of handling workplace stress, was not always intended to be harmful. Indeed, some staff members framed these behaviours as a form of support or safeguarding. However, as Goffman [41] and Link and Phelan [29] argue, stigma is not only about explicit discrimination but also about the subtle social processes that reduce a person’s status. Nevertheless, even under the guise of protection, it serves to diminish PSWs perceived competence and reinforces the assumption that mental health histories or indeed, ongoing mental health states equate to professional inadequacy [4, 42].

Overt forms of stigma were also evident from their colleagues, including exclusion from physical workspaces and team meetings. Such exclusion accentuates the organisational barriers that can marginalise PSWs within the systems they are meant to enhance. Similar patterns of exclusion have been identified in previous studies, where PSWs were denied full team membership or lacked access to the information needed to perform their roles effectively [19, 26]. These practices not only disrupt service continuity but also send a symbolic message about who is considered a “real” or “credible” professional to patients and service-users, reinforcing issues of power dynamics [24]. Perhaps the most explicit form of stigma encountered was where PSWs’ mental health was used to delegitimise their actions or perspectives. This is particularly problematic, as it undermines one of the foundational principles of peer support: the belief that recovery is possible and that lived experience is a form of valuable expertise [12].

### Navigating occupational stigma

In response to these experiences of stigma, PSWs took it upon themselves to navigate the stigma in various ways. PSWs frequently relied on demonstrating commitment and competence to legitimise their roles and gain the respect of colleagues, especially where there was a lack of support [43]. While this strategy can effectively counteract stigma by challenging stereotypes of unreliability or fragility [44], it simultaneously reinforces unequal power dynamics, placing the responsibility on PSWs rather than institutions to validate their worth [24]. This form of compensatory labour is often seen among stigmatised professionals who feel compelled to “earn” their place within an organisation [45, 46]. This continual performance of credibility can produce emotional strain, overwork, and burnout, outcomes that paradoxically mirror the very vulnerabilities PSWs are employed to address [47].

Second, PSWs actively endorsed the value of their lived experience, positioning it as a form of “invaluable expertise” that traditional professionals may lack. This reclaiming of lived experience as an asset rather than a liability represents a powerful form of resistance to stigma. It also reflects broader efforts within the recovery movement to reframe mental health narratives around capability and contribution rather than deficiency within mental health systems [38, 48]. By foregrounding the insights gained through personal recovery, PSWs challenge the dominant biomedical paradigms that have historically positioned individuals with lived experience as passive recipients of care rather than credible knowledge holders [49]. Such repositioning also reflects broader currents within the recovery movement, which seek to reconstruct mental health narratives around agency, capability, and contribution rather than deficiency or pathology [50]. However, this process remains fraught with tension: while lived experience can be a source of expertise, its institutional validation often depends on conformity to professional norms, which may undermine PSWs’ ability to use their lived experience [51]. Thus, PSWs’ efforts to reclaim experiential expertise can simultaneously resist and reproduce aspects of the systems they seek to transform, a paradox that accentuates the complexity of embedding lived experience within professionalised mental health contexts [23, 52]. Indeed, as noted in this study, several PSWs claimed that they were ‘professionals’ as a means of positioning themselves in the workplace hierarchy, and this runs the risk of removing themselves from the true essence of the role [22].

Using the role for reciprocal benefits was another way through which PSWs navigated occupational stigma. By engaging in mutually beneficial relationships, PSWs not only support others’ recovery but also experience affirmation and growth themselves [53]. This mutuality destabilises hierarchical models of care, positioning recovery as a shared, relational process rather than a unidirectional intervention [48, 54]. However, some studies have questioned whether reciprocity should be embedded in the role, as this may blur boundaries [26, 55]. When reciprocity is supported, it can mitigate the isolating effects of occupational stigma and enhance both service quality and PSW wellbeing. However, when it is constrained by boundaries, PSWs may not be able to provide support that utilises their lived experiences and harnesses the transformative potential of the role [56], thereby running the risk of becoming tokenistic symbols of recovery [57].

Thus, careful consideration must be given to how boundaries and reciprocity are managed within the PSW role. Notably, the findings suggest that reciprocal relationships with service-users may function, at least in part, as a response to occupational stigma as a source of affirmation and support when colleagues are perceived as perpetrators of stigma rather than allies. This implies that addressing occupational stigma could itself transform the relational dynamics of the role, such that if PSWs felt genuinely supported by their colleagues, the burden placed on reciprocal relationships with service-users might diminish, allowing those relationships to be grounded in purpose rather than necessity.

### Policy implications

The findings highlight a need for policy frameworks that move beyond rhetorical commitments to “lived experience inclusion” toward the genuine redistribution of power within mental health systems. There needs to be a shift in mindset surrounding negative perceptions ascribed to the PSW role, and instead, focus on how they complement the overall workforce. Policies should explicitly recognise experiential expertise as a distinct and legitimate form of knowledge, embedded in workforce planning, remuneration structures, and leadership pathways. This entails developing clear role definitions, competency standards co-produced with PSWs, and supervision models that safeguard wellbeing.

Furthermore, while this study focused on PSWs’ experiences of occupational stigma, it is worth noting that hierarchical dynamics and occupational devaluation are not unique to PSWs within ward settings. Similar patterns have been observed between registered nurses and healthcare assistants, where rigid role boundaries can reproduce status distinctions and limit collaborative working [58, 59]. As such, this study offers scope for future research where we can learn from other occupations that may experience occupational stigma differently and how they develop alternative or similar strategies to navigate it.

While this study included PSWs from both inpatient and community settings, the data were not analysed by setting, and it is possible that the expression and experience of stigma differs across these contexts. In particular, the risk-averse culture characteristic of inpatient wards, where concerns about patient safety and clinical accountability are heightened, may accentuate hierarchical dynamics and intensify stigmatising behaviours toward PSWs. Future research could usefully explore whether and how the setting moderates the experience of occupational stigma.

## Conclusion

Stigma continues to shape how PSWs are perceived, positioned, and valued within mental health systems. Despite their experiential expertise being increasingly recognised, PSWs often face covert and overt occupational stigma that undermines their legitimacy and restricts the transformative potential of their roles. Through acts of proving themselves, leveraging their experiential expertise and reciprocity, PSWs actively attempt to navigate the stigma they experience in their role. In turn, they attempt to demonstrate that lived experience is not a deficit but a vital form of knowledge that enriches service delivery and recovery practice [38, 53]. Yet, this resistance frequently requires PSWs to engage in continuous self-validation, exposing them to additional emotional and job-related strains. By embedding experiential knowledge within leadership, training, and policy frameworks, mental health systems can move beyond symbolic and tokenistic inclusion toward genuine cultural change, where positioning PSWs as equal partners is important for mental health services going forward and reduces stigma within the system and broader society.

## Data Availability

The dataset generated and/or analysed during the current study are not publicly available due to confidentiality and the sensitive nature of but are available from the corresponding author on reasonable request.

## Abbreviations

PSW: Peer support worker
NHS: National health service
CP: Clinical professional
OP: Organisational manager
SM: Senior manager
